# Dietary Supplements Derived from Food By-Products for the Management of Diabetes Mellitus

**DOI:** 10.3390/antiox14101176

**Published:** 2025-09-26

**Authors:** Ezgi Nur Yuksek, Antia G. Pereira, Miguel A. Prieto

**Affiliations:** 1 Nutrition and Food Group (NuFoG), Instituto de Agroecoloxía e Alimentación (IAA), Universidade de Vigo, Campus Auga, 32004 Ourense, Spain; ezginur.yuksek@uvigo.gal; 2 Investigaciones Agroalimentarias Research Group, Galicia Sur Health Research Institute (IIS Galicia Sur), SERGAS-UVIGO, 36312 Vigo, Spain

**Keywords:** diabetes prevention, insulin resistance, oxidative stress, inflammation modulation, bioactive compounds, clinical efficacy, regulatory framework, functional ingredients

## Abstract

The rising global incidence of diabetes has heightened the demand for prevention strategies that are both effective and environmentally sustainable. In this context, dietary supplements obtained from food processing by-products have emerged as promising candidates, combining high nutritional value with the potential to reduce food waste. These by-products contain abundant bioactive compounds, such as antioxidants, dietary fiber, vitamins, and minerals, that have been associated with improved glycemic regulation. Their beneficial effects are mediated through several interconnected biological mechanisms, including enhanced insulin sensitivity, attenuation of oxidative stress, and modulation of inflammatory pathways. The efficacy of these supplements is supported by findings from preclinical models, clinical trials, and meta-analyses, which also allow comparison with standard antidiabetic interventions. Alongside these findings, considerations related to safety, toxicity, and the regulatory framework are critical for their integration into preventive health strategies. Furthermore, market trends, technological challenges in supplement formulation, and ethical issues surrounding the valorization of food waste are key factors influencing their development and acceptance. Together, these insights underscore the dual therapeutic and ecological potential of food by-product-derived supplements in diabetes prevention, while identifying essential avenues for future research and innovation.

## 1. Introduction

**Figure 1:**
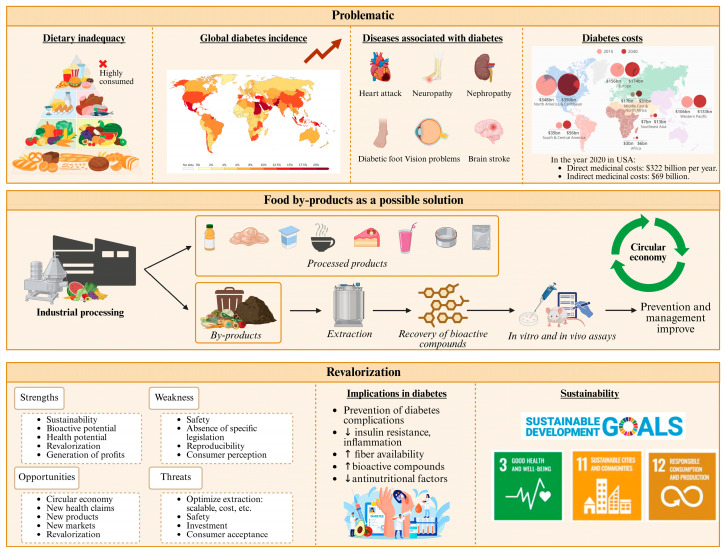
Exploiting food by-products for the development of supplements with antidiabetic activity. Abbreviations. ↓: decrease; ↑: increase.

Diabetes mellitus (DM) is a complex and multifactorial metabolic disease characterized by high blood glucose levels, resulting from an alteration in the secretion or action of insulin, the hormone responsible for regulating the metabolism of carbohydrates, fats, and proteins [1]. DM remains a major global public health challenge, owing to its steadily increasing prevalence in both industrialized and developing countries (Figure 1). Recent estimations from the International Diabetes Federation indicate that nearly 589 million adults aged 20 to 79 are currently living with diabetes worldwide, representing approximately one in nine individuals. This number is projected to rise to 700 million by 2045 and 853 million by 2050, with the increase expected to be more pronounced in urban areas and high-income countries [2,3].

The most frequent forms of this chronic disease are categorized as type 1 diabetes, type 2 diabetes (T2DM) and gestational diabetes. All these types are associated with significant health risks and complications, particularly when not adequately controlled. Such complications include, among others, kidney disease, cardiovascular disorders, neuropathy, and retinopathy, which can substantially impact patients’ quality of life and life expectancy [4]. Under certain conditions, the incidence of these complications is disproportionately high. For example, in T2DM, chronic kidney disease is one of the most prevalent complications, affecting approximately 40% of individuals living with DM and contributing significantly to the global health burden associated with the disease [5].

Therefore, the growing prevalence of diabetes and its complications underscores the necessity of implementing effective prevention and management strategies. Such actions are vital to contain healthcare costs and to address the negative impact on patients’ quality of life [6]. Although conventional pharmacological treatments are effective for glycemic control and prevention of complications, there are inherent limitations, such as side effects, variable adherence, and high costs, which motivate the exploration of alternatives based on functional nutrition and dietary supplements, which have garnered increasing attention due to their modifiable nature and wide accessibility. Specifically, dietary supplements containing bioactive compounds with antioxidant, anti-inflammatory, and insulin-sensitizing effects show significant promise as adjunctive measures in the prevention and management of T2DM [7]. For example, some polyphenols have been shown to improve insulin sensitivity, reduce oxidative stress, and modulate chronic inflammation, key factors in the pathophysiology of T2DM [8]. Furthermore, dietary fiber and other components can positively influence glucose metabolism and the gut microbiota, contributing to improved metabolic regulation [9]. Therefore, dietary interventions with these compounds may help mitigate oxidative stress, reduce chronic inflammation, and improve glucose metabolism, thereby potentially lowering the risk of diabetes onset and slowing disease progression [10,11].

However, despite these promising findings, there remains a lack of data for many dietary supplements regarding critical aspects such as mechanisms of action, pharmacokinetics, and potential toxicity [12]. In fact, current scientific evidence indicates that the efficacy and long-term effects of dietary supplements in diabetes prevention and management remain insufficiently characterized and require further rigorous investigation [13]. It is essential that these investigations consider that diabetes can be caused by a variety of factors, including genetic factors, alterations in insulin production or action, as well as environmental influences and lifestyles that contribute to metabolic dysfunction [1]. This implies that, in some types of diabetes, such as T1DM, which is an autoimmune disease with a fundamentally genetic etiology, the use of nutritional supplements would not have a significant impact on disease prevention, although it could contribute to reducing the risk of developing associated complications. In contrast, in T2DM, the development of which is closely linked to lifestyle, these supplements could play a relevant role in prevention by supporting the control of metabolic risk factors [14,15]. Moreover, diabetes constitutes a heterogeneous metabolic disease, in which metabolic characteristics vary significantly between individuals [16]. Therefore, the metabolism, absorption, and excretion of dietary supplements and bioactive compounds of interest can differ considerably, directly impacting their efficacy and bioavailability [17]. This interindividual variability underscores the importance of adopting personalized approaches to optimize the design and implementation of such nutritional interventions [18]. Furthermore, despite the potential preventive effect attributed to the consumption of these types of supplements, it is essential to consider that these supplements should never replace medical treatment in individuals diagnosed with diabetes. Rather, they should be understood as a complement to conventional therapies, with the potential interaction between supplements and pharmacological treatments carefully evaluated to ensure the safety and efficacy of the therapeutic approach [12].

Integrating such nutrition-based approaches into comprehensive DM prevention programs could contribute substantially to reducing the global burden of diabetes and its related complications [19]. In fact, several studies report that individuals with diabetes are 1.6 times more likely to use this kind of products than the general population [17]. Furthermore, these nutritional alternatives could be accepted by consumers, given their preference and demand for natural products, considered healthier and with a lower risk of side effects [20]. The sustainability of these supplements would be enhanced by the possibility of formulating them from extracts obtained from agro-industrial byproducts, raw material rich in bioactive compounds (e.g., polyunsaturated fatty acids, polyphenols, biopeptides, vitamins, dietary fiber, etc.), thus promoting waste recovery and minimizing environmental impact [21]. This approach contributes to the implementation of circular economy models, in which resources are efficiently reused, reducing waste generation and fostering a more responsible and sustainable production system [22]. In addition, this approach is cost-effective by generating added value to materials that would otherwise represent a cost for the food industry [23].

In this review, the potential contribution of dietary supplements derived from food waste to the prevention and management of diabetes is examined, with particular focus on their mechanisms of action, efficacy, safety profiles, toxicological considerations, and regulatory frameworks. Sustainability, ethical issues, and market dynamics are also explored, offering a multidisciplinary perspective on how the valorization of food waste may support both public health and environmental objectives.

## 2. Review Methodology

This review aims to provide a comprehensive overview of dietary supplements derived from food by-products for the management of diabetes mellitus, with particular attention to studies published since the year 2015. Given the limited availability of clinical studies in this area, earlier experimental research was also considered when relevant. A systematic approach was employed to ensure a thorough and unbiased synthesis of the literature. An extensive search was conducted across major academic databases, including PubMed, Scopus, Google Scholar, and Web of Science, to identify peer-reviewed articles, reviews, books, and conference proceedings relevant to the topic. The search strategy included keywords and combinations such as: “food by-products”, “dietary supplements”, “bioactive compounds”, “diabetes”, “glycemic control”, “in vivo”, “in vitro”, and “clinical trial”.

Studies were included based on their scientific relevance, methodological quality, and alignment with the objectives of the review. Exclusion criteria comprised non-peer-reviewed sources, studies lacking sufficient methodological detail, works not readily accessible, and those unrelated to by-product-derived preparations or their potential antidiabetic effects. A thematic synthesis was performed to identify consistent patterns, highlight research gaps, and provide a critical perspective on the current state of knowledge and directions for future investigation.

## 3. Food By-Products as Sources of Dietary Supplements for Diabetes Prevention

Each year, the food industry produces a significant quantity of materials historically regarded as waste or residues, but now commonly referred to as by-products. These are generated across all phases of the food supply chain, including harvesting, industrial processing, storage, and distribution [24]. Among the most common by-products are fruit and vegetable by-products (e.g., peels, seeds, pulp, bagasse, stems, leaves), products from the meat or fish industry (e.g., bones, spines, viscera), flour and wastewater from food industries, and other remnants that are not part of the final product [25]. All these food by-products are characterized by their richness in bioactive compounds with different biological properties, including their antioxidant, anti-inflammatory, and antitumor capacities [24]. Furthermore, scientific evidence supports the idea that many bioactive compounds exert beneficial effects in the prevention and management of chronic non-communicable diseases, especially T2DM [26]. Among the main compounds identified with antidiabetic properties are polyphenols, dietary fibers, vitamins, minerals, and other phytonutrients of functional interest [27].

Of all bioactive compounds, polyphenols have emerged as the primary focus of scientific research over the past few decades. This class of compounds can be categorized into various subclasses, including flavonoids, anthocyanins, tannins, and phenolic acids [28,29]. These molecules are characterized by reducing oxidative stress, a mechanism that significantly contributes to pancreatic β-cell dysfunction and the development of insulin resistance in individuals with T2DM. They also reduce inflammation through nuclear factor erythroid 2–related factor 2 (Nrf2) activation and nuclear factor κB (NF-Κb) inhibition and inhibit enzymes involved in carbohydrate digestion [30,31]. The bioactive compounds underlying these effects are derived from a diverse array of agro-industrial by-products (Table 1). For instance, grape seeds are an important source of proanthocyanidins and flavonoids [32,33]. Coffee grounds are particularly rich in chlorogenic acids, which have demonstrated hypoglycemic effects through mechanisms such as the inhibition of glucose absorption and the modulation of glucose metabolism [34]. Tea residues, especially from green and black tea, are a relevant source of flavonoids (primarily catechins, theaflavins, and flavanols) which contribute to antidiabetic activity by enhancing insulin sensitivity, regulating glucose uptake in peripheral tissues, and inhibiting key enzymes involved in carbohydrate digestion, such as α-amylase and α-glucosidase [35,36]. In addition to these sources, various underutilized and regionally common plants have also been identified as significant sources of phenolic compounds, including those with antidiabetic activity. These plants include both ornamental species, such as *Camellia japonica*, and wild species, such as *Verbascum* spp. [37,38,39].

Beyond phenolic compounds, dietary fiber also plays a crucial role in metabolic regulation and has been widely studied for its contribution to glycemic control and diabetes management. Dietary fiber with antidiabetic properties is primarily extracted from by-products of fruits, vegetables, cereals, and legumes. This fiber is characterized by its ability to regulate the rate of sugar absorption in the digestive tract, contributing to postprandial glucose control and improving insulin sensitivity [40]. For example, citrus fruit pulp and peel, mango bark and peel, apple peel, wheat bran, onion skin, rice and wheat bran, and corn residue contain pectin, celluloses, hemicelluloses, and lignin, which have demonstrated beneficial effects in regulating glycemia [41,42,43,44]. Other by-products, such as banana peels (Table 1), are able to enhance the activity of key enzymes in the development of T2DM, such as lactase and sucrase [45]. Additionally, certain byproducts rich in fermentable fibers, such as citrus pectin or fructooligosaccharides from roots and tubers, can be used as prebiotics, improving intestinal health and energy metabolism [46].

**Table 1:** Case studies of by-products as a source of antidiabetic compounds.

By-Product	Compounds	Extraction	Concentration	AD Mechanism	Bioactivity	Applications	Ref.
**Polyphenols**							
Orange peel	Naringin, naringenin	MA: EtOH 70%, 72 h	100 mg/kg bw, 4 weeks	↑ insulin, C-peptide, glycogen	AD, AO	PR, DS	[47]
Grapefruit peel	Polyphenols	MA: Acet 80%	80–240 µg/mL	AG	AD, AO, AM, AH	PR, DS	[48]
Lemon peel	Limonoids	MA: EtOH, 24 h	16.53 mg/mL	Join protein	AD, AO	PR, DS	[49]
Pear peel	Rutin, catechin, epicatechin	UAE: MetOH 60%	25–75%	↑ insulin SE, GU	AD, AO, AI	PR, DS	[50,51]
Pomegranate peel	Flavonoids, tannins	SE: MetOH 80%	1 mg/mL	↓ GL	AD, AO, OB	PR, DS	[52]
Orange peel	Flavonoids, phenolics	SE: MetOH 80%	1 mg/mL	↓ GL	AD, AO, OB	PR, DS	[52]
Mango peel	Flavonoids, anthocyanins	Powder	5–10%	↑ insulin SE, GU	AD, AO	PR, DS	[53]
Mango leaves	Polyphenols	SE	80%	AG	AD, AO	CF	[54]
Grape seeds	Flavonoids, procyanidins	SE: EtOH 70%, 3 h	52.01 and 152.18 mg/g dw	AG	AD, AO, OB	PR, DS	[55]
Apple pomace	Quercetin derivates	SE	20 mg/kg	AG	AD, AO	PR, FI	[56]
Carrot pomace	Polyphenols	UAE: 30 °C, 80 min, 500 W	150.6 mg/L	AG	AD, AO	PR, FI	[57]
Walnut husk	Polyphenols	SE	74.08–166.44 mg/g	AG	AD, AO, AM	PR, FI	[58]
**Fiber**							
Banana peel	Dietary fiber	DE, W	nd	↓ FI, GU; ↑ insulin, GLP-1	AD, AO	PR, DS	[59]
Mango peel	Dietary fiber	SE	12.8–23%	↓ FI, GU	AD, AO	PR, FI	[60,61]
Papaya peel	Pectin, lignin	SE: EtOH	nd	↓ FI, GU	AD, MM, SA	PR, FI	[62]
Orange albedo	Pectin	SE: EtOH, 75 °C, 1 h	18.73%	AG	AD, TP	PR, FI	[63]
**Vitamins**							
Orange, grapefruit, and lemon peel	Vitamin C	MA: EtOH, 24 h	110.4, 113.3, and 58.6 mg/100 g	↑ glycemic control	AD, AO	PR, FI	[64]
**Fatty acids**							
Walnut oil cake	Omega-3	SE	52–70%	AG	AD, AO, AI, CP	PR, FI	[65]
Fish waste	Omega-3	UAE: hex, 60 °C, 80 min	45.1%	AG	AD, AI, CP	PR, FI	[66,67]
**Proteins**							
Fish processing waste	Bioactive peptides	EAE: papain, bromelain	18.49%	↑ GLP-1, ↑ insulin SE	AD, AO	PR, FI	[68,69]
Salmon co-products	Bioactive peptides	EAE: FoodPro, alcalase	57–74%	AG	AD, AO	PR, FI	[70]
Cheese by-product	Whey proteins	SE	85%	↑ insulin SE, lipid metabolism	AD, TP	PR, FI	[71]
**Other**							
Tomato waste	Lycopene	UEA: EtOH, 220 W, 25 min	12–19 µg/g dw	↓ GL	AD, AO	PR, FI	[72]
Tomato waste	Lycopene	SE: Acet:hex 1:3	5.32 mg/100 g fw	↓ GL	AD, AO, AI	PR, FI	[73]
Carrot pomace	*β*-Carotene	UAE: 30 °C, 80 min, 500 W	150.58 mg/L	↓ GL	AD, AO	PR, FI	[57]
Carrot pomace	Carotenoids	UAE: EtOH 50%, 40 °C, 750 W	51%	↓ GL	AD, AO, AI	PR, FI	[74]
Orange albedo	Hesperidin	SE: MetOH, RT	1.0–2.8%	↓ GL	AD, AI, CP	PR, FI	[75]

Abbreviations. Extraction. MA: maceration; SE: solvent extraction; DE: decoction; UAE: ultrasound-assisted extraction; EAE: enzyme-assisted extraction; Solvents. MetOH: methanol; Acet: acetone; W: deionized water; hex: hexane. Mechanism. GU: glucose uptake; SE: sensivity; FI: food intake; GL: glucose levels; Bioactivities. AO: antioxidant activity; AI: anti-inflammatory; AM: antimicrobial; AH: anti-hypertensive; OB: anti-obesity; AA: anti-aging; AD: anti-diabetic; AG: α-amylase and α-glucosidase inhibition; CP: cardiovascular protection; MM: microbiota modulation; SA: satiating. Applications. TP: technological properties; PR: preservative; DS: dietary supplementation; CF: commercial formulations; FI: food ingredient; Symbols. nd: not determined; ↑ improve; ↓: decrease; dw: dry weight; fw: fresh weight; RT: room temperature.

Food by-products are also a significant source of essential micronutrients such as vitamins (e.g., D, C, E, B complex) and minerals (e.g., selenium, zinc, chromium, manganese, and potassium) with antidiabetic potential [76]. For instance, vitamin D, which can be effectively extracted from cocoa bean shell or anchovy leftovers [77,78], play functional role on glucose tolerance through its effects on insulin secretion and insulin sensitivity [17]. However, these effects are not consistently observed across all vitamin D-enriched food formulations; the most significant outcomes are achieved when such formulations also include calcium, proteins, and amino acids [16,79]. In the case of vitamins C and E, which can be effectively extracted from by-products of wild apples, citrus fruits, prickly pears, and broccoli [80,81,82,83], their intake has been associated with a decreased risk of developing T2DM by up to 25%. This effect is primarily attributed to their role in enhancing insulin sensitivity and protecting pancreatic β-cell function through the mitigation of oxidative stress [84,85,86]. Conversely, B vitamins, which are predominantly found in significant concentrations in animal-based by-products (e.g., salmon processing residues) [87,88], contribute to glycemic control by reducing oxidative stress, improving endothelial function, and modulating homocysteine levels. In fact, deficiencies in these vitamins have been linked to insulin resistance and increased risk of T2DM and its complications [89,90]. Moreover, these vitamins are also recommended during the use of treatments for T2DM, such as metformin, whose prolonged administration has been associated with vitamin B12 deficiency [91].

Regarding minerals with antidiabetic potential, zinc and selenium are predominantly extracted from by-products generated during the processing of meat, offal, fish, and dairy products [92,93,94]. In addition, whole wheat processing residues represent a valuable source of trace elements such as zinc and chromium, further highlighting the potential of plant-based by-products in the recovery of essential micronutrients [92,93,94]. Manganese is particularly abundant in vegetable and cereal processing wastes, with recovery potentials estimated to provide 25–50% of the recommended daily intake [95]. Potassium is normally extracted for chestnuts by-products [96]. However, it is unclear whether all of potassium formulations are linked to clinically significant increases in dietary potassium [97]. Dietary formulations often incorporate several of these minerals to enhance the product’s potential and benefits. However, although multivitamin and mineral-rich products are used by a large portion of the population in the form of dietary supplements, the effects of these multivitamin and mineral supplements on people with DM have barely been studied. For instance, in studies conducted with rats, it has been observed that diabetes progression in male rats is attenuated by these types of supplements. However, the consumption of these supplements in female rats did not protect against disease progression. This may suggest a gender difference in the pathogenesis of diabetes [98].

In addition to polyphenols, vitamins, minerals, and dietary fiber, which have been widely studied for their antidiabetic effects, there is growing interest in other, less-explored nutrients and bioactive compounds derived from food by-products that show promise in the prevention and management of T2DM. Preliminary studies have demonstrated the potential of protein hydrolysates obtained from animal-based food processing by-products, such as fish skins, bones, and meat trimmings, which may exert insulinotropic and glucose-lowering effects through modulation of incretin hormones and inhibition of dipeptidyl peptidase-IV activity [70,99]. Similarly, bioactive lipids and omega-3 fatty acids extracted from fish and vegetables by-products have shown beneficial effects on insulin sensitivity and inflammatory markers associated with metabolic dysfunction [67,100,101]. Furthermore, certain underutilized plant food wastes, such as fruit peels, seed cakes, and vegetable stalks, contain a variety of phytochemicals and secondary metabolites with antidiabetic potential, including alkaloids or saponins, which may contribute to glycemic control through mechanisms such as glucose uptake enhancement, α-glucosidase inhibition, and antioxidant activity.

In conclusion, food by-products represent an underutilized but extremely promising source of bioactive compounds and nutrients with therapeutic potential. Their use in the formulation of functional dietary supplements could significantly contribute to the prevention and management of T2DM, improving metabolic health through diverse and complementary molecular mechanisms. However, further scientific studies are needed, as well as consumer education courses.

## 4. Mechanisms of Action of Dietary Supplements Derived from Food By-Products Involved in Diabetes Prevention

**Figure 2:**
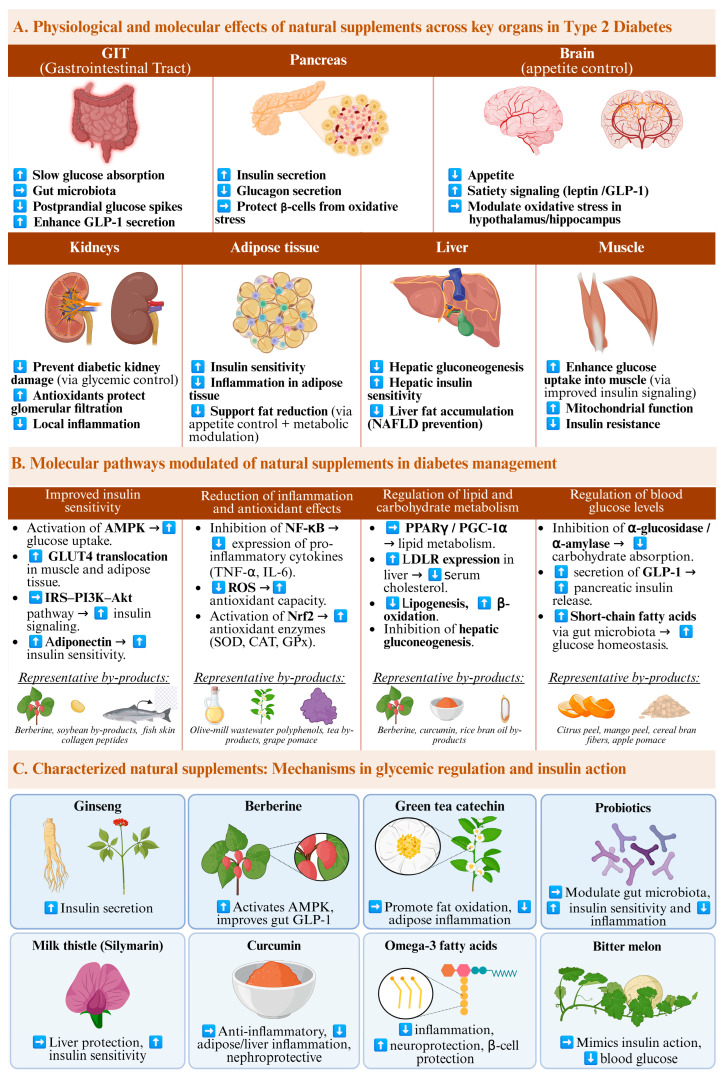
Main mechanism of action of antidiabetic compounds. Abbreviations. ↓: decrease; ↑: increase; →: modulate.

Building upon the extraction and characterization of bioactive compounds from agro-industrial by-products detailed in the preceding section, it is imperative to investigate the molecular mechanisms underlying their antidiabetic properties. These bioactive constituents have been extensively documented for their ability to enhance glucose metabolism, improve insulin sensitivity, and modulate hormones and enzymes involved in glycemic regulation [102]. Their modes of action commonly encompass the stimulation of insulin secretion, attenuation of insulin resistance, and facilitation of glycogen synthesis in the liver [103]. They also modulate carbohydrate-digesting enzymes, such as *α*-amylase and *α*-glucosidase, thereby impeding glucose absorption and attenuating postprandial blood glucose spikes (Figure 2) [104]. Additionally, most of these bioactives exhibit antioxidant and anti-inflammatory properties that protect pancreatic cells from oxidative stress (OS) and mitigate chronic inflammation linked to the progression of diabetes [105]. Some substances, such as fish protein hydrolysates, can stimulate the secretion of gut hormones and suppress appetite. These effects further support weight management and glycemic control [106]. Certain supplements can also contribute to improved metabolic health by modulating gut microbiota [107]. Growing evidence highlights that supplements derived from food by-products offer a promising and multifaceted approach for the prevention and management of diabetes. A comprehensive understanding of the underlying mechanisms is vital not only to validate the therapeutic potential of these bioactive compounds but also to guide their rational incorporation into functional foods, nutraceuticals, or pharmaceutical products. Therefore, this section will systematically explore the principal mechanisms responsible for their antidiabetic effects.

### 4.1. Regulation of Blood Glucose Levels

The onset of DM results in impaired regulation of blood glucose levels, which in untreated individuals typically remain elevated above the normal physiological range. Maintaining glucose within this narrow range is essential for anabolic processes, as anabolism requires tightly regulated blood glucose levels [108]. In healthy individuals, the pancreas serves as a key regulator by secreting insulin to lower blood glucose and glucagon to increase it, thereby maintaining glucose homeostasis. Insulin controls glucose balance by modulating hepatic and renal glucose production, as well as promoting glucose uptake in peripheral tissues, primarily skeletal muscle [109]. However, in individuals with DM, pancreatic function is compromised; specifically, in T2DM, the pancreas exhibits impaired insulin secretion and/or beta-cell dysfunction, leading to inadequate insulin production relative to the body’s needs [110].

Various strategies exist to reduce elevated blood glucose levels in T2DM. One such approach involves inhibiting the action of digestive enzymes responsible for breaking down starches into simple sugars during digestion, such as *α*-amylase [111]. By inhibiting this enzyme, carbohydrate absorption is limited, thereby reducing postprandial glucose spikes [112]. Consequently, insulin demand decreases, leading to more effective blood glucose control. These mechanisms contribute to protecting against insulin resistance and facilitate reductions in body weight and adiposity, two critical factors implicated in the pathogenesis of T2DM. Importantly, these effects can be achieved through the use of various bioactive compounds, as demonstrated in sources such as white kidney bean extract extracted from the *Phaseolus vulgaris* plant [111]. Another example include mangiferin, a xanthone found in mango fruit and its byproducts, that has an inhibitory effect on *α*-glucosidase, the enzyme responsible for hydrolyzing shorter starch chains and disaccharides [113,114].

Another complementary approach focuses on modulating hormone secretion, particularly gut hormones like glucagon-like peptide-1 (GLP-1). GLP-1 increases insulin secretion and suppresses hepatic glucose production; therapies that enhance GLP-1 activity, including GLP-1 mimetics and dipeptidyl peptidase inhibitors, are effective in lowering blood glucose and are inspired by the natural response of the body to nutrient intake and gut microbial signals [115]. Furthermore, regulating the composition and activity of the gut microbiota has emerged as a promising strategy, as a balanced microbiome can influence metabolic pathways, improve insulin sensitivity, and modulate inflammatory responses associated with T2DM [116]. For example, supplementation with prebiotics or specific probiotics has been shown to beneficially alter gut microbiota composition, leading to improved glycemic control in clinical studies [117]. Among the prebiotics evaluated, the use of cinnamon (*Cinnamomum cassia*) stands out as a dietary supplement that has demonstrated efficacy in modulating the intestinal microbiota, with the consequent significant reduction in blood glucose levels (BGL) and HbA1c levels in T2DM patients, particularly those with suboptimal glycemic control (glycosylated hemoglobin (HbA1c) >8%). Significant improvements in HbA1c levels were observed following daily supplementation with 1–6 g of cinnamon for 2 to 4 months. However, these values may vary depending on the populations and starting conditions [12,118].

Another supplement that has been shown to be effective in the treatment of diabetes is chromium-based supplements, which help maintain normal blood glucose levels. However, these effects are only observed in individuals whose T2DM is due to insulin resistance and severe metabolic dysregulation, and are not effective when the condition is due to other physiological causes [119,120,121]. Examples of such supplements include chromium picolinate, an insulin sensitizer that promotes the activation of insulin receptor kinases at the plasma membrane. This activation initiates a signal transduction cascade that results in the translocation of glucose transporter type 4 to the cell surface, thereby amplifying insulin signaling and enhancing glucose uptake [122,123].

### 4.2. Improved Insulin Sensitivity

Insulin resistance is defined as one of the most critical factors in the development of DM [124]. Several studies have highlighted the potential of phenolic compounds to enhance insulin sensitivity. Consequently, vegan, and vegetarian diets, which are abundant in plant-based foods rich in phenolic compounds, have been shown to improve insulin secretion and sensitivity. This improvement is correlated with reductions in HbA1c levels and enhanced *β*-cell function, thereby mitigating insulin resistance [125]. Additionally, they provide protection against reactive oxygen species (ROS), which is critical for managing the inflammatory and they inhibit gluconeogenic enzymes, thereby suppressing gluconeogenesis, processes associated with DM [126].

Fatty acids have also been linked to better insulin sensitivity in both diabetic animals and patients [127]. Omega-3 fatty acids exert beneficial effects on metabolic function, improving insulin sensitivity primarily through anti-inflammatory pathways and the modulation of adiponectin levels. Specifically, *α*-linolenic has been reported to protect pancreatic β-cells and ameliorate diabetic neuropathy, while linoleic acid intake has been associated with a reduced risk of T2DM and improved renal outcomes in some studies [128].

Insulin sensitivity can also be improved through supplementation with certain alkaloids. For example, berberine, an alkaloid extracted from various plant sources such as *Coptis chinensis* and *Verbascum sinuatum*, help regulate blood glucose levels in vitro and in vivo by boosting insulin sensitivity, offering a comparable effect to the drug metformin. At the molecular level, berberine activates AMP-activated protein kinase, which increases GLUT4 translocation to the cell membrane in muscle and adipose tissue, enhancing glucose uptake. It also influences insulin receptor substrate signaling, improving downstream PI3K–Akt pathway activation, which contributes to better insulin signaling. In the liver, berberine upregulates low-density lipoprotein receptor expression at both mRNA and protein levels, promoting cholesterol clearance and linking lipid metabolism to glycemic control. These coordinated molecular actions support its insulin-sensitizing effects, which in some studies have been shown to be comparable to those of metformin. It also lowers HbA1c, as well as fasting and postprandial blood glucose levels [39,129,130].

### 4.3. Reduction in Inflammation and Antioxidant Effects

Diet plays a fundamental role in influencing cytokine concentrations and the inflammatory process, acting as a key mechanism for controlling inflammation. Following a Mediterranean style eating pattern, which includes abundant consumption of olive oil, nuts, fruits, and vegetables, has been shown to reduce levels of inflammatory cytokines [131]. On the other hand, diets that contain high amounts of trans-unsaturated fatty acids and sugars are linked to increased production of these inflammatory molecules. Therefore, dietary recommendations aimed at reducing inflammation emphasize the consumption of fats rich in polyunsaturated fatty acids, particularly omega-3 fatty acids as these nutrients help to lower cytokine levels and thereby decrease inflammation [132,133]. Nutraceuticals containing omega-3 fatty acids act as agonists of peroxisome proliferator-activated receptors (PPAR)-γ and PPAR-*α*, whose activation may alleviate inflammatory responses in DM [128,134]. For instance, the effect of docosahexaenoic acid (DHA)-enriched fish oil on gene expression levels of p53, NF-κB, and (PPAR)-γ was studied in T2DM patients. In this double-blind study, results show how moderate doses of DHA-enriched fish oil supplementation increased PPAR-γ activity. Concretely, authors suggested that the substitution of polyunsaturated fatty acids (PUFAs) as natural ligands for PPAR-γ instead of chemical medicines can lead to the protection of cardiovascular system against atherosclerosis lesion formation [135].

Diet also plays a fundamental role in reducing the production of reactive oxygen species (ROS), which is vital in the management of diabetes, as the disease is associated with a compromised antioxidant defense system [136]. Elevated glucose concentrations provoke mitochondrial dysfunction, which further amplifies ROS generation. Moreover, multiple pathways contribute to ROS formation in DM, including the activation of the polyol pathway, accumulation of advanced glycation end products (AGEs), engagement of AGEs receptors, activation of protein kinase C, and heightened flux through the hexosamine biosynthetic pathway. OS also adversely affects pancreatic *β*-cell performance, diminishing both insulin secretion capacity and insulin quality [133,136,137,138]. Alpha-lipoic acid (ALA) is a well-studied antioxidant that exemplifies the therapeutic potential of such compounds in the management of diabetes. In particular, ALA has been shown to mitigate elevated oxidative stress associated with distal symmetric polyneuropathy, one of the most common complications in diabetic patients [139]. Overall, OS is a major contributing factor of diabetes, as it leads to a decrease in antioxidant enzyme activity and an increase in oxidative DNA damage [140].

### 4.4. Regulation of Lipid and Carbohydrate Metabolism

Various naturally occurring compounds have been investigated for their potential to modulate glucose transporters and carbohydrate metabolism, which play crucial roles in postprandial glucose and insulin responses [141]. These findings have contributed to the development of several patented formulations aimed at regulating lipid and carbohydrate metabolism. For example, mangiferin inhibits two key enzymes involved in glucose metabolism and energy homeostasis: phosphoinositide 3-kinase (PI3K) and serine/threonine protein kinase (Akt) [114]. Another example includes berberine, an alkaloid that targets key regulators of cellular energy homeostasis. It activates AMP-activated protein kinase and modulates the PPARγ co-activator (PGC)-1*α*, pathways involved in both lipid and carbohydrate metabolism. Through these actions, berberine promotes fatty acid oxidation, improves glucose uptake, and regulates hepatic gluconeogenesis, contributing to a balanced metabolic profile and supporting overall energy homeostasis [114]. Curcumin has also been described as a lipoxygenase inhibitor, although its precise mechanism of action is not fully defined. It also exerts lipid-lowering effects by increasing low-density lipoprotein receptor expression at the mRNA and protein levels in the liver [114]. Animal studies indicate that curcumin may improve glucose tolerance partly by stimulating glucagon-like peptide 1 (GLP-1) secretion, thereby enhancing insulin release and glycemic control [142]. At the molecular level, curcumin modulates several signaling pathways relevant to metabolic regulation. It inhibits the NF-κB pathway, reducing pro-inflammatory cytokine production, and activates Nrf2, which promotes antioxidant defense. In addition, curcumin has been reported to activate AMP-activated protein kinase, a central regulator of energy metabolism, thereby promoting fatty acid oxidation and improving insulin sensitivity. Through these combined actions, curcumin exerts effects on both lipid metabolism and carbohydrate utilization, while also protecting β-cells against oxidative and inflammatory stress [143]. Another compound whose effectiveness on lipid metabolism has been investigated in rat studies is 6-gingerol. This study suggests that 6-gingerol may positively influence lipid metabolism by hinder the movement of CD36 (which is a fatty acid transporter involved in lipid homeostasis) from the cytoplasm to the cell membrane, so that normal skeletal muscle would not take up excess fatty acids [144].

## 5. Efficacy of Dietary Supplements Derived from Food By-Products in Diabetes Prevention

The traditional treatment of diabetes has been widely studied in different in vitro, in vivo, and clinical trials, with a special focus on drugs such as metformin and thiazolidinedione pioglitazone (Table 2). Metformin, a guanidine derivative, is one of the primary oral therapeutic options for lowering blood glucose in T2DM as supported by numerous clinical studies [145,146]. Its mechanism of action is based on the induction of glucose uptake to the enterocyte layer of the intestine, which is associated with improved glycemic control [147]. On the other hand, thiazolidinedione pioglitazone acts as an agonist of the peroxisome proliferator-activated receptor gamma (PPARγ), leading to reduced blood glucose levels, improved insulin sensitivity, decreased vascular inflammation, and enhanced lipid profile and endothelial function [148]. In recent decades, the use of these drugs has declined due to potential adverse effects such as weight gain, bladder cancer risk, and decreased bone density. However, they are still widely used in combination with other T2DM therapeutic agents because they are cost-effective, and have regained popularity due to new positive evidence, including cardioprotective effects [149]. However, using pharmacological therapies alone presents several limitations, especially in individuals with T2DM, such as the difficulty in maintaining prolonged behavioral changes, medication side effects, and associated costs that can limit accessibility and adherence to treatment. These limitations underscore the need to explore and develop new complementary strategies that are safe, accessible, and sustainable [150]. In this context, dietary supplements derived from food by-products emerge as a promising alternative, offering potential metabolic benefits along with an ecological and economic approach, warranting further research to effectively integrate them into diabetes prevention programs.

These supplements are typically derived from natural raw materials, often utilizing by-products rich in bioactive compounds [151,152,153]. Among the primary bioactive constituents incorporated into supplement formulations are polyphenolic compounds, dietary fiber, vitamins, minerals, polyunsaturated fatty acids, and amino acids [154]. These compounds have demonstrated the ability to modulate key metabolic pathways, including glucose uptake, insulin sensitivity, oxidative stress, and inflammation. While multiple molecular targets underlying these effects have been proposed, it is essential to characterize the specific mechanisms of action for each individual compound. Moreover, their efficacy, bioavailability, and safety must be confirmed through in vivo studies and rigorously compared to those of conventional antidiabetic therapies. Such evidence is necessary to support their integration into functional foods, nutraceuticals, or pharmaceutical formulations aimed at diabetes prevention.

### 5.1. Polyphenolic Compounds

Different polyphenols have been studied as supplements for the treatment of diabetic related pathologies, showing improvements in different biomarkers mainly associated with anti-inflammatory pathways, although studies focused on these samples remain limited. For instance, a randomized controlled trial with 35 diabetic and overweight patients was assessed (Table 2). After 6 weeks of supplementation with 205 mg/day of a mixture of epigallocatechin gallate, epigallocatechin, epicatechin gallate, and epicatechin extracted from green tea, a significant effect in the weight regulation during a positive energy imbalance was observed [155]. Similar conclusions were obtained in a single-center, placebo controlled, double-blind study, where the supplementation with 530 mg/day of the same catechin mixture to 65 subjects was performed for 8 weeks. Results showed a reduction in the circulation of serum amyloid plasma, that could be explained by the stimulation of fat oxidation via activation of the LKB1/AMP-activated protein kinase pathway or through the modulation of COMT activity [156]. The effect of curcumin supplementation was also assessed in another randomized, double-blind placebo-controlled study, with doses of 1500 mg/day for 10 weeks. Results showed the positive effect of curcumin supplementation in T2DM patients, by reducing fasting blood glucose and weight [157]. In another study, a polyphenol-rich antioxidant supplement containing pomegranate extract, green tea extract, and ascorbic acid was administered to 114 male and female non-smokers (56 in the treatment group and 58 in the placebo group) with T2DM and no complications. The results demonstrated significant antagonistic effects against oxidative stress and lipid peroxidation in these patients, suggesting potential benefits in preventing cardiovascular complications associated with T2DM [158]. Therefore, polyphenols are promising candidates for developing effective adjunct therapies in diabetes management.

### 5.2. Fiber

Randomized controlled trials have shown that viscous dietary fibers can contribute to improved glycemic control and may reduce cardiovascular risk in individuals with diabetes. Clinical studies have reported beneficial effects following supplementation with various fibers such as insoluble fiber, galacto-oligosaccharides, chicory inulin, and beta-glucan. Similarly, animal research has demonstrated positive outcomes on blood glucose levels, HbA1c, and gut microbiota diversity when supplemented with soluble fibers derived from sources like wheat bran, barley, and beta-glucan. However, the effects on insulin sensitivity remain variable. Reviews suggest that prebiotic fibers may support glycemic regulation and gut health, while soluble fibers could also aid in managing insulin and body weight [13,159]. Despite these promising findings, further rigorous studies are necessary to determine the specific benefits of different fiber types and how best to incorporate them into dietary interventions for diabetes.

### 5.3. Vitamins and Minerals

Vitamins and minerals are micronutrients required in small quantities which role is to act as essential coenzymes and cofactors for metabolic and cellular reactions [160]. Their deficiency contributes to the worsening of diabetic pathology [153]. For instance, chromium (Cr) deficiency is linked to insulin signaling impairment, which is a crucial step in glucose regulation [161]. This effect was examined in several clinical studies. In one of them, 32 patients with T2DM received 200 µg/day of Cr, while the other 32 received a placebo. After 12 weeks of administration, results showed that Cr intake leads to lower fasting glucose and insulin levels and improved HOMA-IR levels, suggesting reduced insulin resistance and increased insulin sensitivity. Therefore, Cr supplementation can be considered as an accurate complementation to improve crucial parameters of metabolism, inflammation, oxidative stress, and blood pressure in T2DM patients [162]. Similar results were obtained in another study, where 600 µg/day of Cr supplementation for 4 months showed a reduction in fasting and postprandial glucose in T2DM patients [123].

A similar pattern is observed in the presence of zinc (Zn) deficiency, which has been associated with insulin secretion impairment, insulin sensitivity decrease, and inflammatory biomarkers increment, with studies showing that patients with low Zn blood concentrations are more likely to have had diabetes for a longer time, have poorer glucose control, and have reduced pancreatic *β* cell function [163]. For instance, in a double-blind placebo-controlled study, T2DM patients were administrated with 50 mg/day of Zn for 12 weeks, showing an improvement in fasting glucose and insulin resistance that may be explained due to the effect of this micronutrient in insulin signaling and *β* cells function [164]. Although these studies did not focus on vitamins and minerals present in agricultural byproducts, the presence of these micronutrients in industrial residues makes them a promising strategy to be studied for the development of supplements for diabetic prevention.

### 5.4. Amino Acids and Proteins

The potential benefits of amino acids (AAs) and proteins as supplements for T2DM patients have been supported by various mechanisms. AAs are not only essential substrates for protein synthesis but also play a key role in cellular energy production [165]. Furthermore, dietary protein has been shown to positively influence insulin sensitivity, adiposity, and glucose metabolism. Evidence suggests that protein intakes exceeding 1.2 g/kg/day can lead to improved glycemic control. One contributing factor is that protein metabolism requires relatively low amounts of insulin, whether endogenous or exogenous. In addition, protein consumption promotes greater satiety, which may aid in weight management. Specific AAs, particularly the branched-chain amino acids such as leucine, have been implicated in enhancing both insulin sensitivity and insulin secretion [152]. This body of scientific evidence has led to the development of various AAs-based supplements aimed at managing T2DM. For instance, the effect of AAs supplementation on T2DM patients was assessed in a randomized, single-blind, crossover study involving 65 patients evaluated the effects of essential AAs supplementation versus placebo over a 12-week period. The results demonstrated a positive impact of EAA treatment on glycemic control, with a significant reduction in HbA1c levels compared to placebo [166]. In another study, AAs supplementation of 12 g/day for 42 weeks 65–85-year-old T2DM patients showed a significant decrease on fasting and postprandial glycemic with a reduction in HbA1c. Moreover, it was observed a decrease in fasting insulin levels and HOMA-IR index during AAs supplementation. This study supports that long-term oral amino acid supplementation represents a promising complementary strategy focused on muscle and metabolic health for T2DM elderly patients [167]. These previous studies have used pure commercial AAs for supplementation. Nevertheless, agri-food by-products are known to be rich sources of these AAs. By optimizing extraction techniques, it is possible to obtain a high degree of purification of the AAs of interest [168,169]. As such, utilizing agri-food by-products as a source of AAs may offer a cost-effective and sustainable alternative for nutritional supplementation in diabetic patients, and merits further research.

### 5.5. Polyunsaturated Fatty Acids

PUFAs are integral components of cell membranes, having specific functional, metabolic and signaling roles. Moreover, these compounds are categorized as vital nutrients due to their role in the treatment of non-alcoholic fatty liver, autoimmune reactions, and different chronic diseases [151,170]. Since humans do not possess enzymes to place double bonds in position 3 and 6, these fatty acids (FA) are considered essential. Beyond the most relevant *n*-3 FA, alpha-linolenic acid, eicosapentaenoic acid (EPA), and docosahexaenoic acid (DHA), are specially highlighted, while linoleic and arachidonic acid are the most important *n*-6 FA. *n*-3 essential FA have shown anti-inflammatory, immunomodulatory, and antioxidant effects [171]. Concretely, supplementation of EPA and/or DHA have shown positive modulatory and anti-inflammatory effects, also suggesting providing health benefits to obese, diabetic, or those suffering from cardiovascular diseases [172], but also showing benefits to treat mental disorders such as dementia and depression [173]. However, age, health, and physiological conditions are factors affecting the adequate dose of these compounds, with a daily recommended intake that ranges from 40 to 250 mg [172]. One of the most relevant food byproducts matrices containing an excellent composition of FA are fish by-products, with high amounts of EPA and DHA [67]. Despite the lack of specific studies on the effect of these compounds extracted from fish by-products, there are studies establishing the relationship between the consumption of these compounds and benefits for the prevention or improvement of diabetes symptoms (Table 2). A double-blinded randomized study was carried out to establish the effect of EPA, and DHA supplementation in T2DM patients. This clinical trial was performed for 12 weeks in 100 patients, with doses of 500 mg of EPA and 200 mg of DHA. Results showed how FA supplementation in T2DM patients led to improvements in insulin levels, inflammation markers, antioxidant status, and serum cholesterol levels. However, results also showed no significant effect in homeostatic model assessment of insulin resistance index (HOMA-IR) and quantitative insulin sensitivity check index (QUICKI). This suggests that the potential beneficial effect of these FA supplementation is effective although limited depending on the dose [174]. For instance, in a double-blind controlled study, 56 participants with T2DM were submitted to fish oil supplementation with high content of EPA and DHA Individuals were divided in two groups: fish oil supplementation with 1.8 g EPA, 3.0 g DHA, and 5.9 g total *n*-3 of FA per day for 9 weeks and corn oil with 8.5 g of linoleic acid per day for 9 weeks. Results revealed that high doses of fish oil intake led to a moderate increase in blood glucose and decrease in insulin sensitivity. Moreover, the alteration of fat and carbohydrate utilization was identified in a time-dependent manner [175]. In a different double-blind controlled study, the administration of EPA and DHA in T2DM patients was assessed for 10 weeks. Results showed how 310 mg of EPA and 210 mg of DHA led to a decrease in HOMA-IR levels while QUICK levels were increases, suggesting that the consumption of these FA has a positive metabolic impact by improving insulin sensitivity while decreasing insulin resistance. As a result, T2DM patients had a better glucose metabolism and a reduced burden on pancreatic insulin production [176].

**Table 2:** Compilation of cases of studies regarding the effects of supplements in diabetes and related disorders.

Study	Treatment	Mechanism Suggested	Therapeutic Effect	Ref.
**Traditional treatments**				
DBPC RCT (n = 41)	Met, 1 g, b.i.d., 26 weeks	N.S.	↑ intestinal glucose uptake, glycemic control	[147]
DBPC RCT (n = 366)	Pio, Met, Dapa, 15–30 mg/day, 24 weeks	N.S.	↓ in HbA1c of −0.38 to −0.83%	[177]
**PUFAs**				
DBPC RCT (n = 170)	DHA, 350 mg/day, 2 years	N.S.	Not slowing action in NPDR progression	[178]
RCT (n = 84)	EPA, DHA, 60 + 308 mg/day, 9 months	↓ AA	↑ maternal and fetal FA status (T1DM)	[179]
Mice (n = 56)	EPA, DHA, 1%, 15 days	↓ OS, AI, ┤ *β*-cell apoptosis	Prevent pancreatic injury	[180]
PS (n = 47,663) **	N.S.	AI, ↓ VLDL-C	↓ CHD	[181]
DBPC RCT (n = 26) **	EPA, DHA, PUFA, 1.8, 3.0 and 5.9 g/day, 9 weeks	Change energy use, ↓ glucose uptake	↑ blood glucose; ↓ insulin sensitivity	[175]
DB (n = 100)	EPA, DHA, 500 and 200 mg/day, 12 weeks	AI	↑ insulin levels; ↓ inflammatory markers, Chl	[174]
DBPC RCT (n = 44)	EPA and DHA, 310 and 210 mg/day, 10 weeks	N.S.	↑ QUICKI levels; ↓ insulin, HOMA-IR levels (T2DM)	[176]
**Amino acids**				
RCT (n = 65)	EAA 11.7 g/day//Tyr 0.1 g/day//Cys 0.4 g/day, 12 weeks	N.S.	↑ myocardial dysfunction (T2DM)	[166]
RCT (n = 34)	EAA 7.6 g/day//Tyr 0.1 g/day//Cys 0.3 g/day, 42 weeks	↑ insulin secretion, postprandial glucose	↑ insulin sensitivity, glycemic control (T2DM)	[167]
**Vitamins and minerals**				
DBPC RCT (n = 64)	Cr, 200 μg/day, 12 weeks	N.S.	↑ insulin sensitivity; ↓ insulin resistance (T2DM)	[162]
RCT (n = 71)	Cr, 600 μg/day, 4 months	↑ insulin signaling, GLUT4 translocation	↓ fasting and postprandial glucose; ≈ lipid profile	[123]
DBPC RCT (n = 44)	Zn, 50 mg/day, 12 weeks	↑ insulin signaling; ↓ insulin resistance	↓ fasting glucose and HOMA-IR (T2DM)	[164]
RCT (n = 100)	Vitamin D, 125 μg/day (5000 IU/day), 12 weeks	N.S.	≈ inflammatory and OS (T2DM)	[182]
DBPC RCT (n = 127)	Vitamin D, 100 μg/day (4000 IU/day), 48 weeks	N.S.	≈ Met (T2DM)	[183]
**Polyphenols**				
RCT (n = 35) *	EGCG 120 mg/EGC 60 mg/ECG 25 mg EC; 2 caps./day; 8 weeks	AI	Regulation of weight gain	[155]
DBPC RCT (n = 64) *	EGCG, EGC, EGC, EC, 2 caps 530 mg/day, 6 weeks	↑ FO: LKB1/AMP, modulation COMT	↓ circulating SAA	[156]
DB (n = 100)	Cur, 80 mg nanocurcumin/day, 12 weeks	AI	↑ insulin levels; ↓ inflammatory, Chl	[174]
DBPC RCT (n = 53)	Cur, 1500 mg, 10 weeks	┤ protein replication, AI cytokines; ↑ RMR	↓ fasting blood glucose and weight (T2DM)	[157]

Abbreviations. Study. DBPC RCT: double-blind placebo-controlled randomized controlled trial; RCT: single-blind, placebo-controlled trial; DB: double-blind controlled study; RCT: randomized controlled trial; PS: prospective study; n: number of individuals. Compounds. Met: metformin; Pio: pioglitazone; Dapa: dapagliflozin; DHA: docosahexaenoic acid; EPA: eicosapentaenoic acid; AA: arachidonic acids; Tyr: tyrosine; Cys: cystine; Cr: chromium; EGCG: epigallocatechin gallate; EGC: epigallocatechin; ECG: epicatechin gallate; EC: epicatechin; FA: fatty acids; Cr: chromium; EAA: essential amino acids; Cur: curcumin; N.S.: not specified; Chl: cholesterol. Mechanism: VLDL-C: very low-density lipoprotein cholesterol; SAA: serum amyloid plasma; LKB1/AMP: liver kinase B1/adenosine monophosphate activated protein kinase; COMT: catechol-O-methyltransferase; HOMA-IR: Homeostatic Model Assessment for Insulin Resistance; QUICKI: Quantitative Insulin Sensitivity Check Index; RMR: resting metabolic rate. OS: oxidative stress; AI: anti-inflammatory; FO: fat oxidation; GLUT4: glucose transporter 4. Diseases. CHD: coronary heart disease; NPDR: non-proliferative diabetic retinopathy; T2DM: type 2 diabetic mellitus. Symbols. n: number of individuals in the study; b.i.d.: twice a day; *: extracts form *Camellia sinensis*; **: fish extracts; ↓ reduction; ┤: inhibition; ↑: improve; ≈: not affected.

## 6. Safety and Toxicity of Food By-Product Derived Supplements

The development of dietary supplements from agri-food by-products presents a key challenge: the high heterogeneity of the extracts used as raw materials [184]. Although the final product must meet defined composition standards (Appendix A), extracts from agricultural by-products can exhibit great variability in their chemical profile [185]. This variability is determined by factors such as the type and origin of the by-product, the variety of the base food, the growing conditions, the industrial processing, and the extraction methods used [186,187]. For example, pomegranate peel extracts, rich in ellagitannins, can differ significantly in their toxicological profile depending on the drying method applied (freeze-drying vs. hot-air drying) [188]. Likewise, waste from the wine industry can concentrate contaminants such as heavy metals (lead, cadmium) if their source is not properly controlled [189]. This heterogeneity in raw materials complicates the supplement standardization process, especially in the early stages of development, and hampers toxicological and pharmacokinetic evaluation. Furthermore, the lack of standardization in the preparation of these dietary supplements complicates comparisons across studies and systematic risk assessment, creating uncertainty about their safety for long-term use or in vulnerable populations [190].

To address these problems, it is essential to conduct a detailed phytochemical characterization of the by-products used. However, most preclinical studies reported in the literature do not perform this characterization, which limits replicability and the ability to extrapolate reliable results on the effectiveness and safety of incorporating these extracts into food products. Furthermore, most of the available studies were conducted using in vitro studies or animal models (especially mice) [24,191]. In these models, some of the tested extracts, especially those rich in phenolic compounds or fiber, have been shown to reduce insulin resistance, improve glucose uptake, and modulate inflammatory pathways involved in the pathogenesis of the disease. Therefore, it is necessary to further explore this knowledge, and it is essential to conduct a greater number of in vivo studies in humans, which should focus on analyzing long-term effects.

Another factor to consider when assessing the safety of these products is the significant differences in absorption, metabolism, and excretion between species and individuals, which in some studies could explain the variability of the effects observed after taking dietary supplements [192]. For example, some studies have detected adverse effects on the liver and kidneys in animals at doses that could be achieved in humans with chronic use of certain supplements, such as mango peel extracts rich in mangiferin [193]. In this context, dose customization and individualized monitoring are key aspects not yet adequately addressed in the scientific literature. Therefore, to ensure public health, all these new food products must undergo safety assessments regulated by regional authorities, such as the European Food Safety Authority (EFSA) in the European Union (EU) or the Food and Drug Administration (FDA) in the United States (US). The EFSA approach evaluates toxicokinetics, genotoxicity, toxicity, and reproductive effects. Studies must meet ethical, methodological, and regulatory standards, including GLP and OECD guidelines. Generally, Oral administration is preferred, and botanical ingredients with a long history of safe use may be presumed safe with sufficient data [194,195].

**Figure 3:**
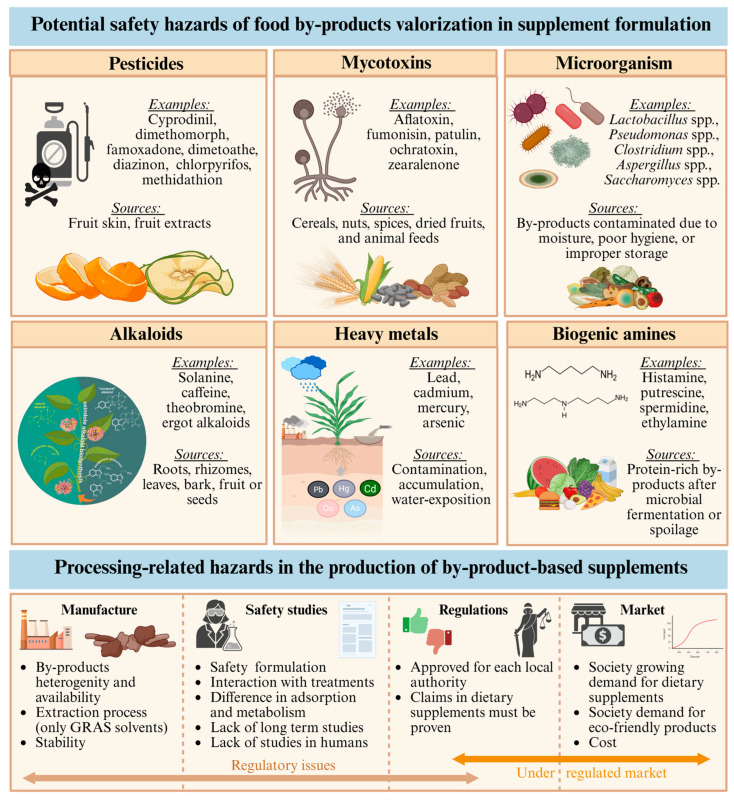
Biological and processing hazards associated with the production of by-product-based supplements.

However, the extracts used in the development of these dietary supplements may contain molecules uncharacteristic of the by-product used such as significant amounts of chemical contaminants such as pesticides and potential pathogens [196]. The primary contaminants typically identified in by-products include pesticides, mycotoxins, microbial agents, heavy metals, alkaloids, and biogenic amines (Figure 3) [194]. In the case of fruits and vegetables, proper processing is essential to prevent the appearance of mycotoxins such as patulin or ochratoxin A, highly nephrotoxic and carcinogenic compounds. Other risks associated with the use of by-products that can compromise product safety include biological instability, potential pathogenic contamination, high water activity, potential for rapid auto-oxidation, and high levels of active enzymes [197]. Most of these problems can be resolved with proper design of the byproduct collection, storage, or processing process to prevent improper handling of byproducts [198].

These biological hazards are associated with serious health risks; consequently, multiple factors must be considered when assessing the suitability of a process for extracting valuable compounds. Accordingly, it is recommended to integrate various types of analyses, such as physicochemical characterization, microbiological assessment, and contaminant detection, on the same food by-product targeted for valorization [199]. In fact, ensuring the quality and safety of these by-products is crucial to fully exploit this valorization strategy [196]. Regulatory bodies such as the EFSA stipulate that foodstuffs derived from valorized by-products must meet established safety and quality standards, ensuring that the resulting products are non-hazardous to health and suitable for human consumption. Similar requirements apply to by-products intended for use in animal feed [200,201]. To meet these requirements, in many cases the extracts are subjected to sterilization, filtration or treatment with absorbent materials before being incorporated into the food matrix [202,203,204]. In other studies, different extraction techniques are used, with the choice of solvent playing a key role. This is because many solvents are toxic, so only those considered GRAS can be used for food purposes. Furthermore, some solvents are characterized by increasing the concentration of the contaminants initially present in the raw material in the extracts [203].

Furthermore, it should be noted that these dietary supplements contain multiple bioactive compounds that can act synergistically to exert a preventive effect against diabetes mellitus type 2 (T2DM). However, these interactions can also increase the risk of toxicity or interference with other pharmacological treatments. For example, extracts rich in flavonoids can inhibit cytochrome P450 enzymes, altering the metabolism of oral antidiabetics such as metformin or sulfonylureas [205]. Likewise, supplements with a high antioxidant content can attenuate the oxidative stress necessary for insulin signaling, paradoxically negatively affecting glycemic homeostasis [206].

Likewise, prolonged use of dietary supplements, even in healthy individuals, could induce cumulative effects. This is because some polyphenols are characterized by low bioavailability but tend to accumulate in tissues, where their long-term function is not yet fully characterized. Furthermore, some studies show that some phenolic compounds exert pro-oxidant effects in cells chronically exposed to these compounds, raising questions about their safety when consumed for months or years without supervision [207,208]. Additionally, although less widespread, the cytotoxicity and mutagenic activity of by-products have also been recently evaluated by in vitro analysis in order to complement physicochemical studies [199], being necessary further investigations. Therefore, it is essential to consider subchronic and chronic toxicity studies that evaluate markers of liver and kidney damage, systemic inflammation, and endocrine disruption in relevant models, as well as pharmacokinetic studies that define parameters such as half-life, metabolism, and excretion of the main components. In this regard, novel strategies such as Food omics in combination with cellular models could be very useful to advance the knowledge of the possible toxicity or risks associated with the consumption of this type of products, since they can provide information about the compound (or compounds) responsible of toxicity and at which level of expression is impacting by applying transcriptomics, proteomics and/or metabolomics techniques [209].

Therefore, although dietary supplements developed from food by-products represent a promising and potentially sustainable strategy for the prevention of T2DM, their practical application requires rigorous evaluation from both toxicological and pharmacological perspectives. Variability in extract composition, the potential presence of contaminants, the lack of standardization of raw materials, and the limited clinical evidence available constitute significant barriers to their validation as safe and effective interventions. Furthermore, it is crucial to evaluate their effects in populations with preexisting conditions or greater biological vulnerability.

## 7. Regulatory Framework

Current legislation on dietary supplements varies significantly across countries. These legislative differences are primarily due to different approaches to risk assessment, the level of state intervention in the market, and the focus on preventive health [210]. This heterogeneity constitutes one of the main challenges for regulating this type of product, especially in a context of globalized trade and the increasing use of digital platforms for distribution [211]. Generally, dietary supplements are regulated as a separate category within food law, although their specific legal treatment depends on the country in question.

**Figure 4:**
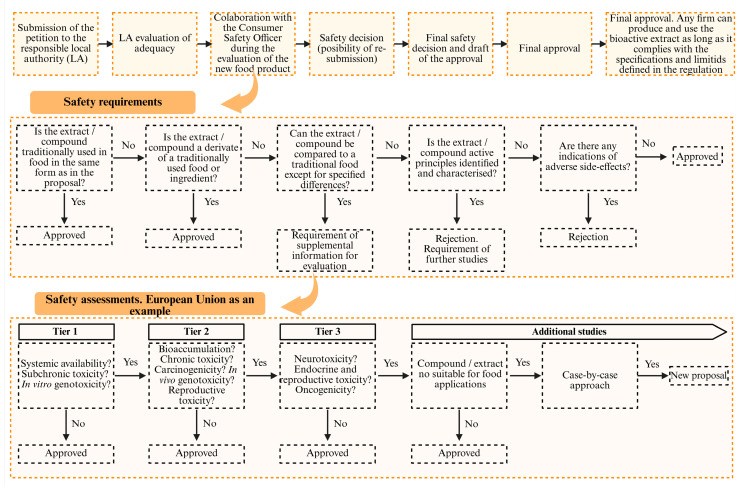
Regulatory framework governing the commercialization of dietary supplements derived from food by-products.

At the European level (Figure 4), most food legislation is based on studies and opinions issued by the European Food Safety Authority (EFSA), the body responsible for providing independent scientific advice on food safety [212,213]. Dietary supplements are covered by Directive 2002/46/EC of the European Parliament and of the Council, which considers them to be products intended to supplement the normal diet, composed of concentrated sources of nutrients or other substances with a nutritional or physiological effect, marketed in measured form [214]. This regulation applies in conjunction with other general food law provisions, such as Regulation (EC) No. 178/2002, which establishes the general principles of food law, and Regulation (EC) No. 1924/2006 on nutrition and health claims made on foods [215]. These regulations also establish the permitted nutritional claims for each food formulation. Only claims approved by the European Commission based on evaluations carried out by the various EFSA panels may be included on product labels. Furthermore, before marketing any of these products, the marketing company must notify the competent authorities in its Member State, which allows for monitoring of these types of products [216,217].

In the United States, another major market for this type of food product, these products are regulated by the Dietary Supplement Health and Education Act (DSHEA) of 1994. This law categorizes dietary supplements as a subcategory of foods, and prior approval by the US Food and Drug Administration (FDA) is not required, except in the case of new, previously unevaluated dietary ingredients [218,219]. Therefore, US legislation is less restrictive, with liability falling on the manufacturer in most cases. However, the FDA, like the EFSA, retains powers in post-market surveillance, including collecting adverse event reports and conducting random inspections. The Federal Trade Commission (FTC), for its part, oversees truthfulness in advertising, introducing a functional division of powers that can complicate the comprehensive oversight of these products [220].

In other countries, different intermediate approaches have been adopted. For example, in Canada, these types of products are classified as natural health products and are regulated by the Natural and Non-prescription Health Products Directorate [221]. This legislation is stricter than that of the United States, as it requires a pre-marketing evaluation phase. As in Europe, this is based on independent reports on the scientific evidence supporting the product’s use, safety, quality, and efficacy. In Japan, dietary supplements are divided into two broad groups: foods for specific health purposes and foods with health claims. Both types of products have different legislation and requirements for approval and marketing.

Therefore, there is a significant legislative gap for these products depending on the country of sale. These regulatory divergences represent one of the major challenges for marketing this type of product due to the trade barriers they create and the increased risk of marketing dietary supplements between countries whose efficacy or safety has not been adequately tested. Various initiatives have been implemented to address this problem, including the Codex Alimentarius, a book that compiles a series of general guidelines in the field of food, which are voluntarily adopted by countries and whose application is not binding [222,223].

Another critical aspect of dietary supplement legislation is labeling control, including nutritional claims. While organizations like the EFSA strictly monitor claims about the beneficial properties of dietary supplements, in practice, it is possible to find products on the market that claim to have health benefits without any solid scientific evidence supporting them. This problem is even more acute in the digital environment, which presents a higher level of food fraud [224,225,226].

In this context, progress in legislation for these products must focus on increasing international cooperation and the development of legal and technological instruments that allow for greater monitoring of quality control and food safety. Likewise, it is essential to develop strategies to improve public awareness of these types of products, so that citizens can critically evaluate the information they receive. This is because the regulation of dietary supplements, far from being a merely administrative aspect, constitutes a key public health tool, which must balance consumers’ right to access safe and effective products with the need to avoid unnecessary risks and deceptive commercial practices.

## 8. Conclusions

The increasing incidence of diabetes demands new, sustainable strategies for prevention and management. Dietary supplements derived from food by-products represent a promising source of bioactive compounds with potential antidiabetic effects. These effects are primarily attributed to improvements in insulin sensitivity, reductions in inflammation, and regulation of glucose metabolism. However, most current evidence is derived from laboratory and animal studies, with relatively few well-controlled human trials. This gap is largely due to variability in bioactive compound content and the lack of standardized dosing.

Among the supplements tested in humans, certain compounds, such as standardized extracts of polyphenols and omega-3 fatty acids, have reached a more advanced stage of development, with evidence from phase 2 clinical trials supporting their potential efficacy and safety. In contrast, many other bioactive compounds reported as antidiabetic agents remain at an earlier stage of investigation and require comprehensive toxicological assessment, pharmacokinetic evaluation, and dose optimization before clinical application can be considered. Additionally, clearer regulatory guidelines are needed to ensure the safety of these supplements. Future research should focus on conducting rigorous clinical trials to verify the benefits of these supplements in humans, while also investigating their interactions with metabolism and the gut microbiota. Improvements in extraction and delivery techniques are expected to enhance their efficacy. Furthermore, close collaboration among researchers, industry stakeholders, and policymakers will be crucial to translate these findings into practical solutions and successfully incorporate these supplements into diabetes management strategies.

## Data Availability

Not applicable.

## Abbreviations

The following abbreviations are used in this manuscript:

Generic.	
Cr	Chromium
DHA	Docosahexaenoic acid
DM	Diabetes mellitus
GLP-1	Glucagon-like peptide-1
HbA1c	Glycosylated hemoglobin
PUFAs	Polyunsaturated fatty acids
BGL	Blood glucose levels
COMT	Catechol-O-methyltransferase
FI	Food intake
FO	Fat oxidation
GL	Glucose levels
GLUT4	Glucose transporter 4
GU	Glucose uptake
HOMA-IR	Homeostatic model assessment of insulin resistance index
LKB1/AMP	Liver kinase B1/adenosine monophosphate activated protein kinase
NF-Κb	Nuclear factor κB
Nrf2	Nuclear factor erythroid 2–related factor 2
OS	Oxidative stress
PPAR	Peroxisome proliferator-activated receptors
QUICKI	Quantitative insulin sensitivity check index
RMR	Resting metabolic rate
ROS	Reactive oxygen species
SAA	Serum amyloid plasma
SE	Sensivity
VLDL-C	Very low-density lipoprotein cholesterol
Applications.	
CF	Commercial formulations
DS	Dietary supplementation
FI	Food ingredient
PR	Preservative
TP	Technological properties
Bioactivities.	
AA	Anti-aging
AD	Anti-diabetic
AG	α-amylase and α-glucosidase inhibition
AH	Anti-hypertensive
AI	Anti-inflammatory
AM	Antimicrobial
AO	Antioxidant activity
CP	Cardiovascular protection
MM	Microbiota modulation
OB	Anti-obesity
SA	Satiating
Compounds.	
AA	Arachidonic acids
AAs	Amino acids
AGEs	Advanced glycation end products
ALA	Alpha-lipoic acid
Chl	Cholesterol
Cr	Chromium
Cur	Curcumin
Cys	Cystine
Dapa	Dapagliflozin
DHA	Docosahexaenoic acid
EAA	Essential amino acids
EC	Epicatechin
ECG	Epicatechin gallate
EGC	Epigallocatechin
EGCG	Epigallocatechin gallate
EPA	Eicosapentaenoic acid
FA	Fatty acids
GLP-1	Glucagon-like peptide-1
HbA1c	Glycosylated hemoglobin
Met	Metformin
N.S.	Not specified
Pio	Pioglitazone
PUFAs	Polyunsaturated fatty acids
Tyr	Tyrosine
Diseases.	
CHD	Coronary heart disease
NPDR	Non-proliferative diabetic retinopathy
T2DM	Type 2 diabetic mellitus
Extraction.	
DE	Decoction
EAE	Enzyme-assisted extraction
MA	Maceration
SE	Solvent extraction
UAE	Ultrasound-assisted extraction
Solvents.	
Acet	Acetone
hex	Hexane
MetOH	Methanol
W	Deionized water
Study.	
DB	Double-blind controlled study
DBPC RCT	Double-blind placebo-controlled randomized controlled trial
n	Number of individuals
PS	Prospective study
RCT	Single-blind, placebo-controlled trial
RCT	Randomized controlled trial

## Appendix A

This table summarizes the minimum quality specifications recommended for extracts derived from food by-products that have demonstrated potential antidiabetic activity. It provides guidance on source identification, extraction methods, composition, dosing considerations, purity and safety parameters, variability, and preliminary toxicity assessment. The aim is to ensure transparency, reproducibility, and safety in the preparation and use of these extracts for research purposes. The checklist is intended as a practical reference to support the standardized reporting and evaluation of bioactive by-product extracts.

**Table A1:** Minimum Quality Specifications for By-product-derived Extracts with Antidiabetic Potential.

Category	Parameter	Specification/Guideline	Notes
Source and Identity	Botanical/by-product identification	Include scientific name, plant part or food by-product, and origin	Ensures traceability and correct identification.
	Extraction method	Detail solvents, temperature, duration, and yield	Extraction method influences chemical composition and safety.
Composition	Marker compounds	Quantify key bioactive compounds (e.g., total polyphenols, fiber)	Allows standardization and comparison between batches or studies.
	Additional constituents	Identify other relevant compounds (e.g., flavonoids, saponins, tannins)	Supports understanding of efficacy and potential toxicity.
Dose Information	Human-relevant dose range	Express as mg/kg body weight or mg/day equivalent	Facilitates translation of experimental data to practical use.
	Unit standardization	Consistently report per extract weight or per active compound	Avoids ambiguity in dosing and reporting.
Purity and Safety	Residual solvents	Follow international guidelines	Prevents exposure to harmful solvents.
	Heavy metals	Below internationally accepted limits	Ensures safety and regulatory compliance.
	Pesticides/Mycotoxins	Below internationally accepted limits	Minimizes health risks from contamination.
	Microbiological quality	Absence of pathogens; total microbial counts within accepted limits	Ensures hygienic quality of extracts.
Variability and Reproducibility	Raw material variability	Document seasonal or batch variations	Accounts for differences in composition between raw material lots.
	Batch-to-batch consistency	Use analytical profiling (e.g., HPLC, LC-MS)	Confirms reproducibility of results.
Toxicity Data	Screening	Include in vitro cytotoxicity and, if available, in vivo safety data	Provides initial safety assessment for potential use.
Regulatory Compliance	Alignment with guidelines	All extracts should comply with the relevant legislation and regulatory requirements of the country in which they are intended to be marketed	Supports safe and regulated use of extracts.
